# Refractory post visual internal urethrotomy bleeding managed by angioembolization

**DOI:** 10.4103/0971-3026.59750

**Published:** 2010-02

**Authors:** Jayesh V Dhabalia, Girish G Nelivigi, Mahendra Singh Punia, Vikash Kumar

**Affiliations:** Department of Urology, K.E.M Hospital, Mumbai, India

**Keywords:** Angioembolization, bleeding, stricture urethra, visual internal urethrotomy

## Abstract

Post visual internal urethrotomy (VIU) bleeding is usually treated successfully with local compression. Angioembolization for post VIU bleeding has not been previously reported to the best of our knowledge. This is a case report of a 55-year-old man who was referred with persistent per urethral bleeding around a Foley catheter, three days following VIU. When standard methods of treatment were unsuccessful, the bleeding was controlled by embolizing the bulbourethral artery with polyvinyl alcohol (PVA) particles.

## Introduction

Visual internal urethrotomy (VIU) using a cold cutting knife is the first choice of treatment for a short-segment stricture of the urethra.[1,2] The most common complications are recurrence of the stricture and bleeding.[2,3] Post VIU bleeding is usually treated with local compression. Rarely, electrofulguration is required. We report a case of post VIU bleeding that failed to respond to local compression and transurethral fulguration. The patient was successfully treated with selective angioembolization.

## Case Report

**Figure 1:**
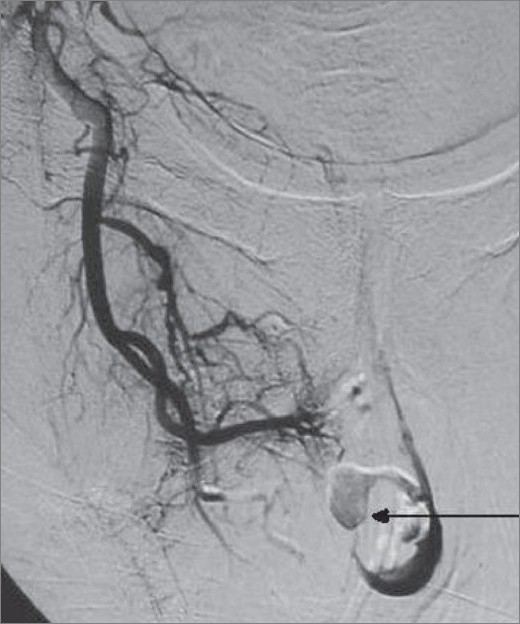
Selective angiography of the bulbourethral artery shows extravasation (arrow) from the bulbourethral artey

**Figure 2:**
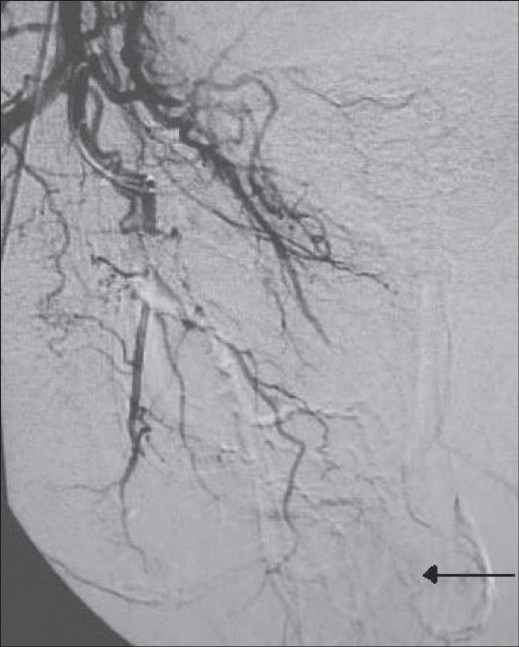
Post angioembolization image shows cessation of extravasation (arrow)

A 55-year-old man was referred with persistent per urethral bleeding around an 18 French Foley catheter, three days after VIU, performed for a 1-cm long mid-bulbar stricture. There was also history of a VIU performed 10 years earlier. The patient's hemoglobin level had dropped from 12 gm% to 4 gm%. All other hematological and biochemical parameters, including the coagulation profile, were normal. USG revealed a normal upper tract. There was a 3 × 2.5 cm bladder clot. The prostate was normal, with a volume of 16 cc. Urethrocystoscopy revealed mucosal edema and a spurter at the 12 o'clock position. Fulguration was done with electrocautery and complete hemostasis was achieved. However, the bleeding recurred after 24 hours and did not respond to local compression. Hence the patient was considered for selective internal pudendal artery angiography, which revealed extravasation from the bulbourethral artery at the distal end of the internal pudendal artery [Figure 1]. Using the transfemoral approach, a 5F sheath was placed in the right femoral artery and a 5F Cobra catheter was advanced to the origin of the internal pudendal artery. A microcatheter-wire system was subsequently advanced into the major bleeding bulbourethral artery, which was embolized using polyvinyl alcohol (PVA) particles (average size 500-750 μm) (Cook Bloomington Inc., USA). The bleeding stopped completely [Figure 2] and the catheter was removed after three days. At three months follow-up, the patient had normal voiding and a normal erection, with no recurrence of bleeding.

## Discussion

VIU using a cold cutting knife at the 12 o'clock position[1] is the universally accepted treatment for a short-segment stricture of the urethra. Electrosurgical treatment of the stricture is not preferred due to a high recurrence rate. Next to recurrence, bleeding is the most common complication.[2,3] The usual position of the bulbourethral arteries is at the 3 o'clock and 9 o'clock positions. However, variable but symmetric location of the bulbourethral arteries as also loss of symmetry in patients with strictures have been reported.[4] The 12 o' clock position, however, remains the most common site for the urethral incision in VIU.

Post VIU bleeding is usually well controlled with local compression and rarely requires electrofulguration. We have shown that in the presence of significant loss of blood and failure of conservative treatment and transurethral fulguration, selective angiography and angiembolization can be used to successfully control bleeding without causing failure of erection or impotence.
